# Mediterranean Diet Effect: an Italian picture

**DOI:** 10.1186/1475-2891-10-125

**Published:** 2011-11-16

**Authors:** Elena Azzini, Angela Polito, Alessandro Fumagalli, Federica Intorre, Eugenia Venneria, Alessandra Durazzo, Maria Zaccaria, Donatella Ciarapica, Maria S Foddai, Beatrice Mauro, Anna Raguzzini, Lara Palomba, Giuseppe Maiani

**Affiliations:** 1 National Institute for Food and Nutrition Research, Via Ardeatina 546, 00178 Rome, Italy

**Keywords:** overall diet quality, oxidative stress, markers of inflammation, Mediterranean dietary pattern

## Abstract

**Background:**

The purpose of this study was to evaluate the overall diet quality effects, mainly on antioxidant nutritional status and some cytokines related to the cellular immune response as well as oxidative stress in a healthy Italian population group.

**Methods:**

An observational study was conducted on 131 healthy free-living subjects. Dietary intake was assessed by dietary diary. Standardised procedures were used to make anthropometric measurements. On blood samples (serum, plasma and whole blood) were evaluated: antioxidant status by vitamin A, vitamin E, carotenoids, vitamin C, uric acid, SH groups, SOD and GPx activities; lipid blood profile by total cholesterol, HDL cholesterol, LDL cholesterol, triglycerides; total antioxidant capacity by FRAP and TRAP; the immune status by TNF-α, and IL-10 cytokines; the levels of malondialdehyde in the erythrocytes as marker of lipid peroxidation.

**Results:**

The daily macronutrients intake (g/day) have shown a high lipids consumption and significant differences between the sexes with regard to daily micronutrients intake. On total sample mean Mediterranean Diet Score (MDS) was 4.5 ± 1.6 and no significant differences between the sexes were present. A greater adherence to a Mediterranean dietary pattern increases the circulating plasma levels of carotenoids (lutein plus zeaxanthin, cryptoxanthin, α and β-carotene), vitamin A and vitamin E. The levels of endogenous antioxidants were also improved. We observed higher levels in anti-inflammatory effect cytokines (IL-10) in subjects with MDS ≥ 6, by contrast, subjects with MDS ≤ 3 show higher levels in sense of proinflammatory (TNF α P < 0.05). Lower levels of MDA were associated with MDS > 4. Our data suggest a protective role of vitamin A against chronic inflammatory conditions especially in subjects with the highest adherence to the Mediterranean-type dietary pattern.

**Conclusions:**

Mediterranean dietary pattern is associated with significant amelioration of multiple risk factors, including a better cardiovascular risk profile, reduced oxidative stress and modulation of inflammation.

## Background

The Mediterranean Diet (MD), a nutritional model inspired by the traditional food regimes of countries in the Mediterranean basin, including Italy, Greece, Spain and Morocco, has entered into the Intangible Cultural Heritage of Unesco. The Mediterranean dietary pattern can be identified in high consumption of fruit and vegetables, olive oil as principal source of fat, low consumption of meat and dairy products and moderate consumption of wine.

The diet should afford an adequate contribution of nutrients to meet the metabolic requirements of individual and to give the consumer safety, quality and varied diet.

The MD seems to provide a balanced diet, suitable for all ages and it is thought to reduce significantly the risk of chronic diseases [1].

Several investigations [2-6] have indicated that this diet is a possible means of protection against cardiovascular problems, metabolic disorders, certain cancers and other age-related degenerative diseases. Several studies have evidenced the correlation between the MD and longevity [7-9]. Sofi et al. [10] have shown that a greater adherence to the MD is associated with a significant improvement in health status, as seen by a significant reduction in overall mortality (9%), mortality from cardiovascular diseases (9%), incidence of mortality from cancer (6%), and incidence of Parkinson's disease and Alzheimer's disease (13%). Benetou et al. [11] have summarized that adherence to the traditional MD is associated with markedly and significantly reduced incidence of overall cancer, which is appreciably larger than predicted from examining individual MD components. Even if Bogani et al. [12] have related MD benefits with the protective role of phenolic diet components, reducing daily oxidative stress, the healthy dietary style effects are related to the overall diet quality. Furthermore, increasing evidence suggests that diet may also be important in modulating inflammation [13]. On the other hand availability and access to a variety of foods have been identified as key elements with psychological processes at individual and social levels and other factors that influence food choices. However, availability and access to food are influenced by socio-economic aspects and prevailing lifestyles. Nowadays a modern Western diet, complete with large amounts of meat, highly processed foods and sweets has become far more common in Italy, especially among the younger generations. So, the MD appears as a valid model of sustainability from the health point of view, helping to support quality in food and in the meantime helping to promote sustainable resource management through environmental sound farming systems linked to territorial characterisation and to local cultural heritage.

The purpose of this study was to evaluate the overall diet quality effects, mainly on antioxidant nutritional status and some cytokines related to the cellular immune response as well as oxidative stress in a healthy Italian population group.

## Methods

### Subjects

Study participants included 300 subjects, aged 20-40 years, living in Southern and Central Italy. The following criteria were considered: no vegan or vegetarian regimes, BMI range from 18.5-25 kg/m^2^, absence of acute or chronic diseases or metabolic disorders, smoking habits, alcohol consumption (< 30 g/d for men and < 20 g/d for women), no drugs (aspirin, anti-inflammatory medications) and no vitamin or mineral supplements. At enrolment, qualified interviewers administered dietary and lifestyle questionnaires to participants. A full medical history, including drug use, was collected. A complete clinical check-up was also performed and participants with pathological diseases, which can present a risk for the volunteers, were excluded. The subjects' heights and body weights were measured.

After this screening only 164 subjects were considered eligible for the study; only 131 of them agreed to undertake the blood collection (table 1). The study was conducted in accordance with the Declaration of Helsinki on the human trial performance and informed consent was provided by participants.

**Table 1:** Characteristics of subjects by sex (mean ± sd)

	Men	Women	P values
n°	64	67	

Age (yrs)	34 ± 4	31 ± 6	0.000

Weight (kg)	80.2 ± 10.0	59.0 ± 8.1	0.000

High (cm)	177.8 ± 7.4	162.1 ± 6.4	0.000

BMI (kg/m^2^)	25.2 ± 2.4	22.5 ± 2.7	0.000

Statistic: Anova

### Study design

In all enrolled volunteers were evaluated: lifestyle; anthropometric measurements (weight, height and circumferences); food consumption by a dietary diary over four consecutive days including the weekend. On blood samples (serum, plasma and whole blood) were assessed: antioxidant status by vitamin A, vitamin E, carotenoids, vitamin C, uric acid, SH groups, SOD and GPx activities; the lipid blood profile by total cholesterol, HDL cholesterol, LDL cholesterol, triglycerides; total antioxidant capacity by FRAP and TRAP; the immune status by TNF-α, as a prototype of Th1 cytokine and IL-10, as prototypes of Th2 cytokine; the levels of malondialdehyde in the erythrocytes as marker of lipid peroxidation.

### Lifestyle evaluation and anthropometric measurements

Previously trained interviewers have administered a questionnaire to all subjects with the aim to provide information on lifestyle, socio-economic levels, physical exercise, smoking habits and alcohol consumption.

Standardised procedures were used to make anthropometric measurements according to Lohman TG [14]. Height was measured to the nearest 0.1 cm with a wall stadiometer Holtain. Body weight was recorded to the nearest 0.01 kg using a calibrated computerized digital balance (K-Tron P1-SR), each participant was barefoot and lightly dressed. The Body Mass Index (BMI) was derived by the ratio of weight in kilograms divided by the square of the height in metres (kg/m2). Arm circumference, waist and hip were measured using a flexible inelastic tape to the nearest 0.1 cm. Waist circumference alone or in relation to hip circumference was used as an index of the distribution of adipose tissue and visceral obesity, as indicator of cardiovascular risk factor [15].

### Food consumption assessment

The food consumption was detected by a validated food diary on four consecutive days including weekend. All consumed foods and beverages were recorded by participants and the day after a dietician verified and checked the registration propriety. Furthermore to improve the accuracy on the estimation of the portions was used a photo album [16]. Italian Food Composition Tables were used to calculate energy, macro and micronutrients from daily consumption [17].

### Mediterranean Diet Score (MDS)

The degree of adherence to the traditional MD was assessed using the Mediterranean Diet Score (MDS) proposed by Trichopoulou A. et al [18]. According to the proposed method, food items were grouped into major food groups based on the Mediterranean diet and for comparison purposes the food items intake (gram/day) was adjusted to 2500 kcal/day for men and 2000 kcal/day for women. Foods groups (vegetables, fruits, legumes, cereals, fish, meat, dairy products, alcohol) as well as relationship between monounsaturated and saturated fatty acids were considered. The Score (MDS) ranged from 0 (minimal adherence to the MD) to 9 (maximum adherence to MD). Three main classes of MDS were arbitrarily identified corresponding to three levels of diet quality from: 0-3 Low Quality, 4-5 Medium Quality and 6-9 High Quality.

### Sample collection, treatment and analyses

Fasting blood samples were collected into vacutainers without anticoagulant or containing EDTA and/or heparin. Plasma and serum were immediately separated by centrifugation and aliquots were stored at -80° C until the analysis. Erythrocyte samples were treated and/or stored at -80°C until malondialdehyde (MDA), SOD and GPx analyses were performed. Precision and reproducibility of measurements were monitored by using pooled human plasma or serum or a multi-parameter control for quantitative clinical chemistry determinations (Clin Chem Control 1, Sentinel Diagnostics, Milan, Italy). Total cholesterol, HDL cholesterol, LDL cholesterol triglycerides and uric acid concentrations were measured using enzymatic tests (Sentinell Diagnostics, Milano, Italy). Total Antioxidant Capacity (TAC) was evaluated by Ferric Reducing Antioxidant Power (FRAP) [19] and by Total Radical-Trapping Antioxidant Parameter (TRAP) [20]. The spectrophotometric assay for measuring thiol groups was based on DTNB or Ellman reagent which reacts with SH groups leading to the formation of a coloured solution which shows a maximum absorption at 412 nm [21]. A two-step sandwich immunoassay ELISA was used for identifying an immune Th1 pro-inflammatory response (TNF-α) or a TH_2 _anti-inflammatory response (IL-10). Activities of erythrocyte antioxidant enzymes were also evaluated by measuring activities of superoxide dismutase (SOD) and glutathione peroxidase (GPx) using ELISA, as well as the contents of erythrocyte malondialdehyde (MDA) by commercial kit purchased from Oxis Research. The determination of plasma retinol, α-tocopherol and carotenoids (lutein plus zeaxanthin, criptoxanthin, lycopene, α and β carotene) concentrations were carried out by high-performance liquid chromatography techniques described by Maiani et al. [22]. Total ascorbic acid was extracted using the method described by Margolis et al [23] and the quantitative analysis was performed using an HPLC system equipped with a coulometric detector (ESA model 580; Chelmsford, MA, USA) [24]. Our laboratories collaborate to the Fat Soluble Vitamins Measurement Quality Assurance Program in human serum and plasma by National Institute of Standard Technology USA.

Statistical comparisons were carried out with analysis of variance (ANOVA) and Student's t-test.

Pearson's and Spearman's correlation coefficients were used to study the correlation between biochemical parameters and Mediterranean Diet Score and Mediterranean Diet Score classes. P-values of 0.05 or less were considered significant.

## Results and Discussion

Physical Characteristics of studied sample by sex are reported in table 1. Mean age was 34 ± 4 years for men and 31 ± 6 years for women, BMI values (kg/m^2^) were 25.2 ± 2.4 and 22.5 ± 2.7 for men and women respectively.

Table 2 and 3 show the daily intakes (g/day) of macronutrients and micronutrients by sex. Daily consumptions have shown an high lipids intake, representing the 38% of total energy while the 44% of total energy was due to the carbohydrates. The percentage of energy provided by fats was higher than LARNs recommendations (25-30%) (daily assumption of nutrient levels for the Italian population) [25], while the energy from carbohydrate was lower than recommended one (55-60%), in addition there was no significant difference between the sexes, except for proteins (%) (P = 0.02) and alcohol (%) (P < 0.000).

**Table 2:** Daily macronutrients intake (g/day) and energy (%) by sex (mean ± s.d.)

	Men	Women	P values
**Energy (Kcal/day)**	2701 ± 469	1971 ± 457	0.000
**Proteins (g/day)**	105 ± 23	75 ± 20	0.000
**(% en)**	16 ± 3	15 ± 2	0.02
**Lipids (g/day)**	112 ± 22	85 ± 23	0.000
**(% en)**	38 ± 5	39 ± 5	n.s
**PUFA (g/day)**	8 ± 3	6 ± 2	0.000
**(% en)**	3 ± 1	3 ± 1	n.s
**MUFA (g/day)**	42 ± 13	31 ± 11	0.000
**(% en)**	14 ± 4	14 ± 4	n.s
**SFA (g/day)**	21 ± 6	17 ± 5	0.000
**(% en)**	7 ± 2	7 ± 2	n.s
**PUFA/SFA**	0.42 ± 0.14	0.42 ± 0.12	n.s
**MUFA/SFA**	2.1 ± 0.6	2.1 ± 0.5	n.s
**Carbohydrates (g/day)**	316 ± 79	234 ± 65	0.000
**(% en)**	44 ± 6	44 ± 6	n.s
**Fiber (g/day)**	17 ± 7	14 ± 6	0.009
**Alcohol (g/day)**	12 ± 13	4 ± 7	0.000
**(% en)**	3 ± 3	1 ± 3	0.000

Statistic: Student's t-test

**Table 3:** Daily micronutrients intake by sex (mean ± s.d.)

	Men	Women	P value
**Iron (mg/day)**	12.6 ± 3.5	9.5 ± 3.3	0.000
**Calcium (mg/day)**	867 ± 328	713 ± 212	0.002
**Potassium (mg/day)**	3067 ± 968	2370 ± 743	0.000
**Phosphorus (mg/day)**	1496 ± 342	1131 ± 283	0.000
**Zinc (mg/day)**	12.3 ± 3.5	8.8 ± 2.7	0.000
**Thiamin (mg/day)**	1.2 ± 0.5	1.1 ± 0.6	n.s
**Niacin (mg/day)**	19.9 ± 5.8	16.0 ± 8.0	0.002
**Vitamin B6 (mg/day)**	2.1 ± 0.7	1.8 ± 1.0	0.05
**Folic acid (μg/day)**	257 ± 68	200 ± 73	0.000
**Riboflavin (mg/day)**	1.7 ± 0.5	1.5 ± 0.8	n.s
**Retinol Eq (μg/day)**	1355 ± 866	1071 ± 616	0.000
**Vitamin E (mg/day)**	15.3 ± 5.2	12.5 ± 4.8	0.002
**Vitamin D (μg/day)**	2.1 ± 1.7	1.6 ± 1.3	n.s
**Vitamin C (mg/day)**	142.8 ± 74.0	114.0 ± 68.0	0.022

Statistics: Student's t-test

Moreover, our findings have shown statistically significant differences (table 3) between the sexes with regard to daily micronutrients intake, also the average intake levels of some micronutrients (iron, calcium and potassium) was not sufficient to meet requirements in women [25].

Finally, it was evaluated the relationship between quality of diet, lifestyle habits and nutritional status. On total sample mean Mediterranean Diet Score (MDS) was 4.5 ± 1.6 and no significant differences between the sexes were present. Table 4 shows the average consumption of food groups by MDS classes. The 26% of subjects had a low quality diet (MDS≤3), the 46% of the sample showed a medium quality diet (MDS = 4-5), while 28% of subjects had a high quality diet (MDS≥6). With increasing classes of MDS, as expected, we observed an augmented consumption of MD typical foods and a reduced intake of MD non-typical foods. In particular, a greater adherence to traditional MD (MDS ≥ 6) was significantly associated with higher consumption of vegetables (P = 0.0008), fruits (P < 0.0000) and fish (P < 0.005) and a lower consumption of meat (P = 0.02), milk and dairy products (P = 0.002), alcohol, although not statistically significant and a better relationship between MUFA and PUFA (P = 0.00003). Non-significant correlation coefficients were found between MDS or MDS classes and a specific biochemical variable, confirming how overall food intake interacts with other dietary and non-dietary factors in a freely-selected diet.

**Table 4:** Average consumption of food groups* (g/day) by MDS classes.

	DIET QUALITY			
	Low (MDS≤3)	Medium (MDS 4-5)	High (MDS≥6)	P values
**%**	26	45	29	
**Cereals (g/day)**	225 ± 53	248 ± 71	267 ± 76	0.02
**Vegetables (g/day)**	167 ± 113	225 ± 127	261 ± 97	0.0008
**Legumes (g/day)**	7 ± 19	18 ± 27	22 ± 23	0.02
**Fruits (g/day)**	162 ± 162	209 ± 188	389 ± 238	0.00000
**Fish (g/day)**	39 ± 40	61 ± 62	88 ± 96	0.005
**Meat (g/day)**	161 ± 112	119 ± 65	114 ± 90	0.02
**Milk and dairy products (g/day)**	305 ± 160	227 ± 128	195 ± 170	0.002
**Alcohol (g/day)**	9 ± 11	8 ± 12	5 ± 10	n.s.
**MUFA/SFA ratio**	1.8 ± 0.4	2 ± 0.5	2.3 ± 0.6	0.00003

*Food groups were adjusted to 2500 kcal for men and 2000 kcal for women

Statistic: Anova

The table 5 describes as lifestyle and sociodemographic factors affect diet quality; in particular a high diet quality and a lower prevalence of cardiovascular risk, calculated on waist circumference values (men ≥ 94 cm; women ≥ 80 cm), have been highlighted.

**Table 5:** Sample distribution (%) by lifestyle habits, sociodemographic characteristics, coronary heart disease risk and diet quality

	DIET QUALITY		
	Low (MDS≤3)	Medium (MDS 4-5)	High (MDS≥6)
	%	%	%
**Smokers**	11	36	16
**No smokers**	81	55	68
**Past smokers**	8	9	16
			
**Socioeconomics**			
**Medium-low**	19	47	34
**High**	40	42	19
			
Cardiovascular risk*	46	39	22

* calculated on waist circumference values: men ≥ 94 cm; women ≥ 80 cm

Lipidemic profile, antioxidant status, immune status and lipid peroxidation indicator by diet quality are reported in table 6. Regarding lipidemic profile, our results have shown that subjects with high consumption of fruit and vegetables have a significant contribution of MUFAs and an increase in plasma LDL-cholesterol levels. So, although LDL are prone to oxidation, in subjects exhibiting a high quality diet with higher consumption of fruits and vegetables, the bioavailable amount of bioactive molecules from the diet seems to promote their protective and preventive action to lipid oxidation against oxidative stress [26-29] and the observed low levels of MDA indicate that body lipids are preserved in larger proportion. The influence of plasma cholesterol levels on the atherosclerotic process and the effects of altering cholesterol levels by diet and other lifestyle behaviours on the progression and development of cardiovascular disease have been reported [30]. Several researchers [31,32] have also suggested that dietary patterns may have an effect on the mechanisms of atherosclerotic plaque vulnerability and the progression to thrombosis. Many studies have demonstrated the inhibitory activity of several compounds, existing in fruits and vegetables, on the oxidation of LDL in vitro [33-35].

**Table 6:** Lipidemic profile, antioxidant status, immune status and lipid peroxidation indicator by diet quality (mean ± SEM)

	DIET QUALITY		
	Low (MDS≤3)	Medium (MDS 4-5)	High (MDS≥6)
**Total cholesterol (mmol/L)**	4.55 ± 0.10	4.63 ± 0.08	4.60 ± 0.10
**HDL- cholesterol (mmol/L)**	1.26 ± 0.05	1.32 ± 0.05	1.35 ± 0.05
**LDL- cholesterol (mmol/L)**	2.95 ± 0.10	3.00 ± 0.10	3.10 ± 0.10
**Triglycerides (mmol/L)**	0.78 ± 0.06	0.77 ± 0.05	0.72 ± 0.06
			
**FRAP (μM)**	976 ± 42	883 ± 22	953 ± 28
**TRAP (μM)**	842 ± 34	855 ± 22	886 ± 26
**Uric acid (mmol/L)**	0.34 ± 0.02	0.35 ± 0.01	0.38 ± 0.02
**SH (mM/l)**	546 ± 31	524 ± 29	520 ± 32
**GPx (UI/gr Hb)**	41.2 ± 2.5	48.8 ± 2.1	49.2 ± 2.7
**SOD (U/l)**	37.0 ± 3.7	41.3 ± 3.6	40.0 ± 5.1
			
**Vitamin A (μmol/L)**	1.87 ± 0.08	1.89 ± 0.06	2.05 ± 0.08
**Vitamin E (μmol/L)**	23.22 ± 0.93	23.44 ± 0.46	24.15 ± 0.70
**Vitamin C (μmol/L)**	49.97 ± 2.27	52.24 ± 1.70	48.27 ± 2.27
**Lutein +Zeaxantin ((μmol/L)**	0.57 ± 0.05	0.63 ± 0.04	0.64 ± 0.05
**Cryptoxanthin ((μmol/L)**	0.16 ± 0.02	0.15 ± 0.02	0.20 ± 0.02
**Lycopen (μmol/L)**	0.79 ± 0.02	0.83 ± 0.02	0.83 ± 0.02
**α-carotene (μmol/L)**	0.07 ± 0.02	0.07 ± 0.01	0.08 ± 0.01
**β-carotene ((μmol/L)**	0.55 ± 0.07	0.57 ± 0.06	0.58 ± 0.07
			
**TNF-α (pg/ml)**	42.4 ± 9.0^a^	35.7 ± 7.0^a^	14.8 ± 2.9^b^
**IL10 (pg/ml)**	8.3 ± 3.3^a^	9.98 ± 1.3^a^	19.5 ± 5.2^b^
			
**MDA (μM/l)**	118 ± 7	104 ± 7	110 ± 7

Statistic: Anova P < 0.05 a versus b

Interesting results appear to underline how circulating plasma levels of carotenoids (lutein plus zeaxanthin, cryptoxanthin, lycopen, α and β-carotene), vitamin A and vitamin E have reached highest values in subjects with a greater adherence to a MD (MDS ≥ 6). Plasma carotenoids may be considered as biomarkers of fruit and vegetable intake by reflecting, at least qualitatively, short-term carotenoid intake. All components of vitamin E, a group of fat-soluble compounds that include both tocopherols and tocotrienols, exist naturally in the MD. The antioxidant activity of vitamin E is derived primarily from α-tocopherol and γ-tocopherol, of which α-tocopherol is most biologically active and the predominant form found in blood, the concentrations of α-tocopherol in human blood are generally four times higher than those of γ-tocopherol, in contrast γ-tocopherol has been found to be more effective than mixed tocopherol in protecting against certain specific types of oxidative damage. We assessed vitamin E status by α-tocopherol plasma levels representing the most widely used biomarker to evaluate it. So an high quality diet consumption (MDS>6) seems to be associated to an improvement of exogenous antioxidants that could exert a better protection against oxidative and nitrosative stress by influencing the *in vivo *oxidant/antioxidant balance.

Same trend was observed regarding to endogenous antioxidants: higher levels were associated with a MDS >4.

**Figure 1:**
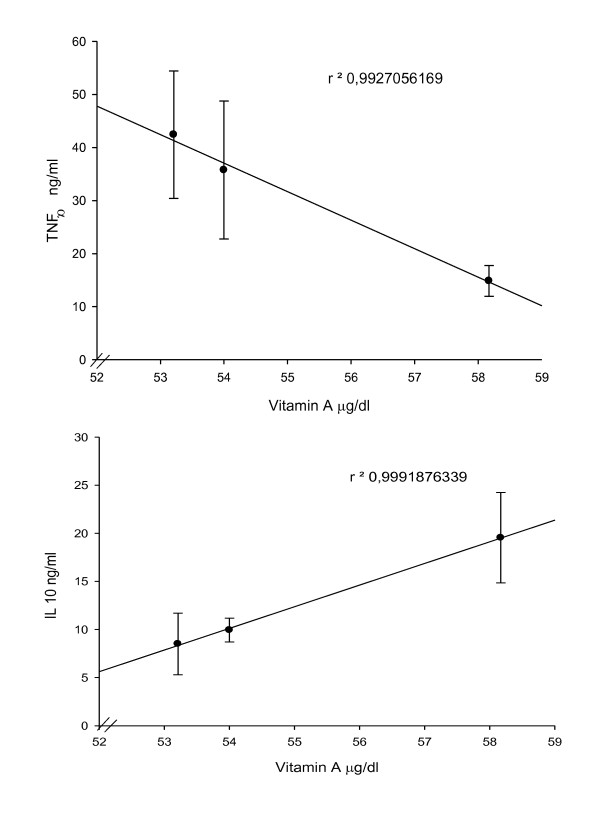
**Levels of TNF α and IL10 versus Vitamin A levels by MDS**.

About the evaluation of immune response we observed higher levels in anti-inflammatory effect of cytokines (IL-10) in subjects with MDS ≥ 6, by contrast, subjects with MDS ≤ 3 show higher levels in sense of proinflammatory (TNF α P < 0.05). In addition Figure 1 shows the levels of TNF α and IL10 versus vitamin A levels by MDS. The analysis of variance was nearby to the significance P = 0.08 and P = 0.06 for TNF α and IL10 respectively. Finally lower levels of MDA were associated to MDS >4.

These findings seem to support the hypothesis of a modulation of the redox status by exogenous antioxidants, in fact, the healthy subjects on a freely-selected diet with a low adherence to MD have demonstrated pro-inflammatory cytokines levels significantly higher than subjects with higher consumption of fruit and vegetables and high MDS, which also show a significant rise in immune status. Even if the exact relation is not clear, as well known, markers of inflammation are linked to chronic diseases, diet is linked to inflammation and a Mediterranean diet pattern is rich in beneficial anti-inflammatory nutrients. Recent studies show that vitamin A can lower the production of pro-inflammatory cytokines such as TNF-α and IL-12 and promote the activation of lymphocyte subpopulations that, through the secretion of specific cytokines (eg IL-10), have the function to return levels of inflammation to the baseline [36]. Our data therefore seem to suggest this trend in the protective role of vitamin A against chronic inflammatory conditions especially in subjects with the highest adherence to the MD. On the other hand, the adherence to the MD, with higher consumption of fruits and vegetables rich in phytochemicals, demonstrates to increase circulating levels of endogenous and exogenous antioxidants, improving immunity and protecting the selected sample from oxidative stress and maintaining a healthy status.

As widely documented, fruit and vegetables consumption has been closely associated with a lower risk of degenerative diseases [37-41].

By a number of international health authorities the phytochemical components of fruits and vegetables were indicated as powerfully beneficial for human health. Differently from other food components, phytochemicals can exert their bioactivity without reaching the systemic circulation. Scarcely-absorbed antioxidants might reach the large bowel contributing to protection from oxidative damage-induced gastrointestinal diseases [42,43]. Dietary habits can play a key role in regulating the redox status of human plasma improving the defense against oxidative damage. The measure of antioxidant capacity reflects the synergistic action of all the antioxidants present in plasma and body fluids, thus providing an integrated parameter rather than the simple sum of measurable antioxidants [20], even if the assay does not account for in vivo antioxidant enzyme activities [44]. The effect that diet exerts on total plasma antioxidant capacity has been over the last decade, the source of much debate. The total antioxidant capacity of body fluids, especially plasma, can be used as an index of redox status of the human organism in both healthy individuals and in those suffering from different diseases [45]. The total antioxidant capacity of biological samples can also be evaluated in clinical studies which measure end-products of free-radical damage of endogenous compounds such as lipids or DNA. Changes from the base-line levels of these products could then be ascribed to changes in the antioxidant capacity of the diet. The antioxidant capacity of biological samples can be monitored by a variety of simple, non-specific, high-throughput screenings assays, which do not necessarily reflect the human physiological mechanisms in vivo [46]. However, studies showing the effect of the global quality diet are lacking. Our results show that subjects with high adherence to MD model had higher levels of plasma antioxidant capacity than those with low MD model indicating that the quality of the diet could play and have a different effect by using a single food matrix. These results confirm those reported by other authors [47,48]. Even if pigments and other phytochemicals could contributed to total antioxidant activity our results have shown that subjects upon high diet quality consumption exhibited an increase in total antioxidant capacity and a decrease in MDA, as well as a decrease in TNFα and an increase of IL10. In addition total antioxidant capacity values correlate with its major small-molecule contributors (urate, ascorbate and α-tocopherol).

Our research represents an Italian picture of healthy subjects on a freely-selected diet and although our data were based on a small number of subjects, the results were in line with the hypothesis that a Mediterranean dietary pattern is associated with significant amelioration of multiple risk factors, including a better cardiovascular risk profile, reduced oxidative stress and modulation of inflammation [49-51].

## Conclusion

In conclusion, the present study shows that the synergistic effects of bioactive food constituents and improving immune system seem to explain the overall diet quality value as well as the increased consumption of fruits and vegetables as focal point of MD. Even if the diet represents only one aspect of healthy lifestyle, the Mediterranean diet seems to be an excellent choice to achieve significant health benefits.

## List of abbreviations

MD: Mediterranean Diet; MDS: Mediterranean Diet Score; SOD: superoxide dismutase; GPx: glutathione peroxidase; HDL: high density lipoprotein; LDL: low density lipoprotein; FRAP: Ferric Reducing Ability of Plasma; TRAP: Total Radical-Trapping Antioxidant Parameter; TNF-α: Tumour Necrosis Factor-alpha; IL-10: interleukin-10; MDA: malondialdehyde; BMI: body mass index; MUFA: monounsaturated fatty acid; PUFA: polyunsaturated fatty acid; SFA: saturated fatty acid.

## Competing interests

The authors declare that they have no competing interests.

## Authors' contributions

GM designed the research and organized the data collection; EA, PA and GM wrote the paper; EA, AF, FI, AD, MZ, DC, MSF, BM and AR conducted the research; EA analysed the data; LP collaborated to the outline of the study and to the final review of the manuscript.

All authors read and approved the final manuscript.
